# Different curcumin forms selectively bind fibrillar amyloid beta in post mortem Alzheimer’s disease brains: *Implications for* in-vivo *diagnostics*

**DOI:** 10.1186/s40478-018-0577-2

**Published:** 2018-08-09

**Authors:** Jurre den Haan, Tjado H. J. Morrema, Annemieke J. Rozemuller, Femke H. Bouwman, Jeroen J. M. Hoozemans

**Affiliations:** 1 Department of Neurology, Amsterdam Neuroscience, VU University Medical Center Alzheimer Center, Mailbox 7057, 1007 MB Amsterdam, the Netherlands; 2 Department of Pathology, Amsterdam Neuroscience, VU University Medical Center, Amsterdam, the Netherlands

**Keywords:** Curcumin, Amyloid-beta, Alzheimer’s disease, Cerebral amyloid angiopathy (CAA), Neurodegeneration, Biomarker, Immunohistochemistry

## Abstract

The combined fluorescent and Aβ-binding properties of the dietary spice curcumin could yield diagnostic purpose in the search for a non-invasive Aβ-biomarker for Alzheimer’s disease (AD). However, evidence on the binding properties of curcumin, its conjugates and clinically used bio-available formulations to AD neuropathological hallmarks is scarce. We therefore assessed the binding properties of different curcumin forms to different neuropathological deposits in post-mortem brain tissue of cases with AD, other neurodegenerative diseases, and controls. Post mortem brain tissue was histochemically assessed for the binding of curcumin, its isoforms, conjugates and bio-available forms and compared to routinely used staining methods. For this study we included brains of early onset AD, late onset AD, primary age-related tauopathy (PART), cerebral amyloid angiopathy (CAA), frontotemporal lobar degeneration (FTLD) with tau or TAR DNA-binding protein 43 (TDP-43) inclusions, dementia with Lewy bodies (DLB), Parkinson’s disease (PD) and control cases without brain pathology. We found that curcumin binds to fibrillar amyloid beta (Aβ) in plaques and CAA. It does not specifically bind to inclusions of protein aggregates in FTLD-tau cases, TDP-43, or Lewy bodies. Curcumin isoforms, conjugates and bio-available forms show affinity for the same Aβ structures. Curcumin staining overlaps with immunohistochemical detection of Aβ in fibrillar plaques and CAA, and to a lesser extent cored plaques. A weak staining of neurofibrillary tangles was observed, while other structures immunopositive for phosphorylated tau remained negative. In conclusion, curcumin, its isoforms, conjugates and bio-available forms selectively bind fibrillar Aβ in plaques and CAA in post mortem AD brain tissue. Curcumin, being a food additive with fluorescent properties, is therefore an interesting candidate for in-vivo diagnostics in AD, for example in retinal fluorescent imaging.

## Introduction

Aggregation and extracellular deposition of the amyloid-beta (Aβ) peptide in amyloid plaques is a pathological hallmark and an early event in the pathophysiology of Alzheimer’s disease (AD) [4, 18]. Currently AD diagnosis can be made with help of clinical criteria with the support of measurements of Aβ_1–42_ and tau_− 181_ in cerebrospinal fluid (CSF), amyloid-positron emission tomography (PET), cortical atrophy on magnetic resonance imaging (MRI), or hypo-metabolism by fluorodeoxyglucose–PET (FDG-PET) [35]. As these are all either invasive or time consuming investigations, a patient friendly and repeatable amyloid or Aβ biomarker is urgently needed for diagnosis as well as therapy monitoring in clinical trials [15]. Curcumin is a dietary spice that has been shown to have combined fluorescent and Aβ-binding properties [16, 37, 39]. Therefore, curcumin has the potential to be used as a tool in a non-invasive Aβ-directed diagnostic assay for AD. A recently published paper by Koronyo et al. shows possible application of curcumin for in-vivo detection of retinal amyloid [22].

Curcumin is a polyphenolic compound derived from the root *Curcuma longa* or turmeric*,* traditionally used in Indian cuisine and Ayurvedic medicine. Turmeric naturally contains three curcuminoids namely; curcumin (≈77%), demethoxycurcumin (DMC)(≈17%) and *bis-*demethoxycurcumin (BDMC)(≈3%) [10]. Curcumin is fluorescent by nature [16, 37]. It has anti-carcinogenic, anti-inflammatory, anti-oxidant and anti-angiogenic properties and binds to Aβ and interferes with Aβ aggregation [10, 28]. More specifically curcumin binds Aβ-oligomers and fibrils in vitro [41, 52], binds plaques in APP_SWE_/ PS1_ΔE9_, Tg2576 and 5xFAD transgenic mice [23, 29, 30, 53] and binds Aβ-plaques in post mortem human AD brain tissue [23, 39, 49, 53]. Two reports also described moderate binding of curcumin to neurofibrillary tangles in AD brain tissue [36, 39].

In order to apply curcumin as a tool for in-vivo detection of Aβ its poor bio-availability and in-vivo metabolism should be considered. The majority (35–89%) of the orally administered curcumin is present in the feces, as the intestinal mucosa and mucus form a physical barrier [14, 43]. Secondly, curcumin that does reach the circulation undergoes phase 1 (reduction) and phase 2 metabolism (conjugation) in the liver. Reductases reduce curcumin to dihydrocurcumin, tetrahydrocurcumin, hexahydrocurcumin etc. [31, 32, 42]. In addition, curcumin is conjugated to sulfates and glucuronides [5, 17, 31, 32]. Therefore, the majority of circulating curcumin is conjugated. To overcome curcumin’s poor bio-availability, nano-particulate drug delivery systems have been developed for oral administration including micelles, solid lipid nanoparticles and liposomes (Theracurmin™ [20, 40, 44], Novasol™ [7, 21, 45], Longvida™ [11, 22]).

So far, there are no reports on the Aβ-binding properties of conjugated curcumin or bio-available forms of curcumin. Insight into binding properties of circulating curcuminoids in the blood (e.g. DMC, BDMC, and curcumin conjugates) and specific bio-available formulations is needed for the design of biomarker assays using oral (bio-available) curcumin formulations. In this study, we assessed the binding properties of curcumin, its isoforms, its conjugates and clinically used bioavailable curcumin supplements to neuropathological hallmarks of AD and other neurodegenerative features in post mortem brain tissue.

## Materials and methods

### Post mortem brain tissue

Post mortem brain tissue was obtained from the Netherlands Brain Bank (NBB; Amsterdam, the Netherlands). Prior to death, donors signed informed consent for brain autopsy and use of brain tissue and medical records for research purposes. For this study we selected 5 early onset AD (EOAD)-patients, 5 late onset AD (LOAD) patients, 5 AD cases with capillary cerebral amyloid angiopathy (CAA type-1), 5 controls, 5 cases with primary age related tauopathy (PART), 3 frontotemporal lobar degeneration (FTLD) cases with tau pathology (FTLD-tau), 1 FTLD case with TDP-43 pathology (FTLD-TDP), 2 Parkinson’s disease (PD) cases and 1 dementia with Lewy bodies (DLB) case who donated their brains to the NBB between 2000 and 2014. EOAD cases had a reported disease onset before 65 years of age (mean age of death 61.6 years), while LOAD cases had reported disease onset > 65 years. Neuropathological diagnosis of AD was made following the NIA-AA criteria including Thal-staging for Aβ stage, Braak-and-Braak-staging for NFTs and CERAD-staging for neuritic plaques [1, 38, 48]. Pathological diagnosis of CAA-type 1, FTLD (tau and TDP-43), DLB and PD were made following Thal-, Mackenzie-, McKeith- and Braak-staging criteria respectively [2, 25–27, 33, 34, 47]. Two of the FTLD-Tau cases were P301L mutations (chromosome 17), while one was a sporadic Pick’s disease case. The FTLD-TDP43 case was a progranulin mutation. See Table 1 for cohort characteristics.

**Table 1:** Cohort characteristics

#	PM delay (h:min)	Pathological diagnosis (mutation)	Sex	Age	Braak	Amyloid
1	6:35	Control	F	92	III	0
2	7:10	Control	F	78	I	A
3	4:35	Control	F	78	II	A
4	7:15	Control	M	95	II	B
5	5:15	Control	M	83	I	A
6	5:00	EOAD	M	61	VI	C
7	5:05	EOAD	M	59	VI	C
8	4:40	EOAD	M	62	V	B
9	4:45	EOAD	M	64	V	C
10	5:15	EOAD	M	62	VI	C
11	5:30	LOAD	M	88	VI	C
12	7:00	LOAD	F	92	V	C
13	4:40	LOAD	F	89	V	C
14	6:25	LOAD	F	91	IV	C
15	3:05	LOAD	M	74	VI	C
16	6:05	CAA type 1	M	68	II	C
17	4:20	CAA type 1	M	81	V	C
18	4:20	CAA type 1	F	96	V	C
19	3:25	CAA type 1	M	94	V	C
20	6:00	CAA type 1	F	75	V	C
21	5:30	PART	F	81	II	0
22	3:52	PART	F	89	III	0
23	5:00	PART	F	87	II	0
24	5:50	PART	F	93	II	0
25	6:35	PART	F	103	IV	0
26	3:35	FTLD-TDP (Progranulin)	F	76	n.a.	A
27	5:23	FTLD-Tau (P301L)	M	60	n.a.	0
28	6:25	FTLD-Tau (P301L)	M	64	n.a.	0
29	6:15	FTLD-Tau (PiD)	M	60	n.a.	0
30	24:00	PD	M	57	I	0
31	33:00	LBD/AD	F	78	VI	B
32	14:00	PD	M	68	I	0

Abbreviations: *CAA type 1* capillary cerebral amyloid angiopathy type 1, *EOAD* early onset Alzheimer’s disease, *FTLD-tau* frontotemporal lobar degeneration with tau pathology, *FTLD-TDP-43* frontotemporal lobar degeneration with TDP-43 pathology, *LOAD* late onset Alzheimer’s disease, *n.a.* not applicable, *PART* primary age-related tauopathy, *PiD* Pick’s disease, *PM delay* post-mortem delay and *PD* Parkinson’s disease

### Immunohistochemistry (IHC)

For each case we assessed formalin-fixed paraffin-embedded tissue sections (5 μm thick) from mesencephalon, hippocampus, frontal or occipital lobes. Paraffin-embedded tissue sections were deparaffinized and rehydrated using xylene, alcohol and phosphate-buffered saline (PBS; pH 7.4). Endogenous peroxidase activity was blocked by 0.3% H_2_O_2_-treatment of the sections. Heat induced antigen retrieval was performed in 0.01 M citrate buffer pH 6.0 using an autoclave. Sections were incubated overnight with primary antibody and subsequently washed with PBS (see details in Table 2). Primary antibodies were detected using EnVision (Dako, Glostrup, Denmark) followed by washing in PBS. Antibody complex binding was visualized using 3,3′-diaminobenzidine (DAB) and sections were counterstained with hematoxylin. Stained sections were covered using Quick-D (Klinipath, Beek, the Netherlands).

**Table 2:** Primary antibodies used for immunohistochemistry and fluorescent staining

Antibody	Source	Species	Dilution
IC-16	Carsten Korth, Heinrich Heine University Dusseldorf, Düsseldorf, Germany [50]	Mouse	1:400
4G8	Biolegend, San Diego, CA, USA	Mouse	1:8000
AT8	Invitrogen, Thermo Fisher Scientific, MA, USA	Mouse	1:800
pTDP-43	Cosmo Bio, Tokyo, Japan	Mouse	1:8000
LB-509	Zymed, Thermo Fisher Scientific, MA, USA	Mouse	1:200

### Thioflavin-S staining

Thioflavin-S staining was performed by incubating deparaffinized and rehydrated sections with 1% thioflavin-S (Sigma-Aldrich) solution (demineralized water) for 1 min, followed by rinsing off excess thioflavin-S using 70% alcohol. Following staining slides were washed using PBS and covered using Tris-buffered saline (TBS)/glycerol mounting medium.

### Curcuminoid staining

Curcuminoids, conjugates and bio-available forms of curcumin were dissolved in 0.5 M NaOH to a stock concentration of 20 mg/ml (see details in Table 3). Final concentrations of 0.2, 0.1 and 0.05 mg/ml were prepared by dilution in PBS. Deparaffinized and rehydrated sections were incubated with curcumin solutions for 10 min, followed by washing in PBS. Stained sections were covered using TBS/glycerol mounting medium.

**Table 3:**
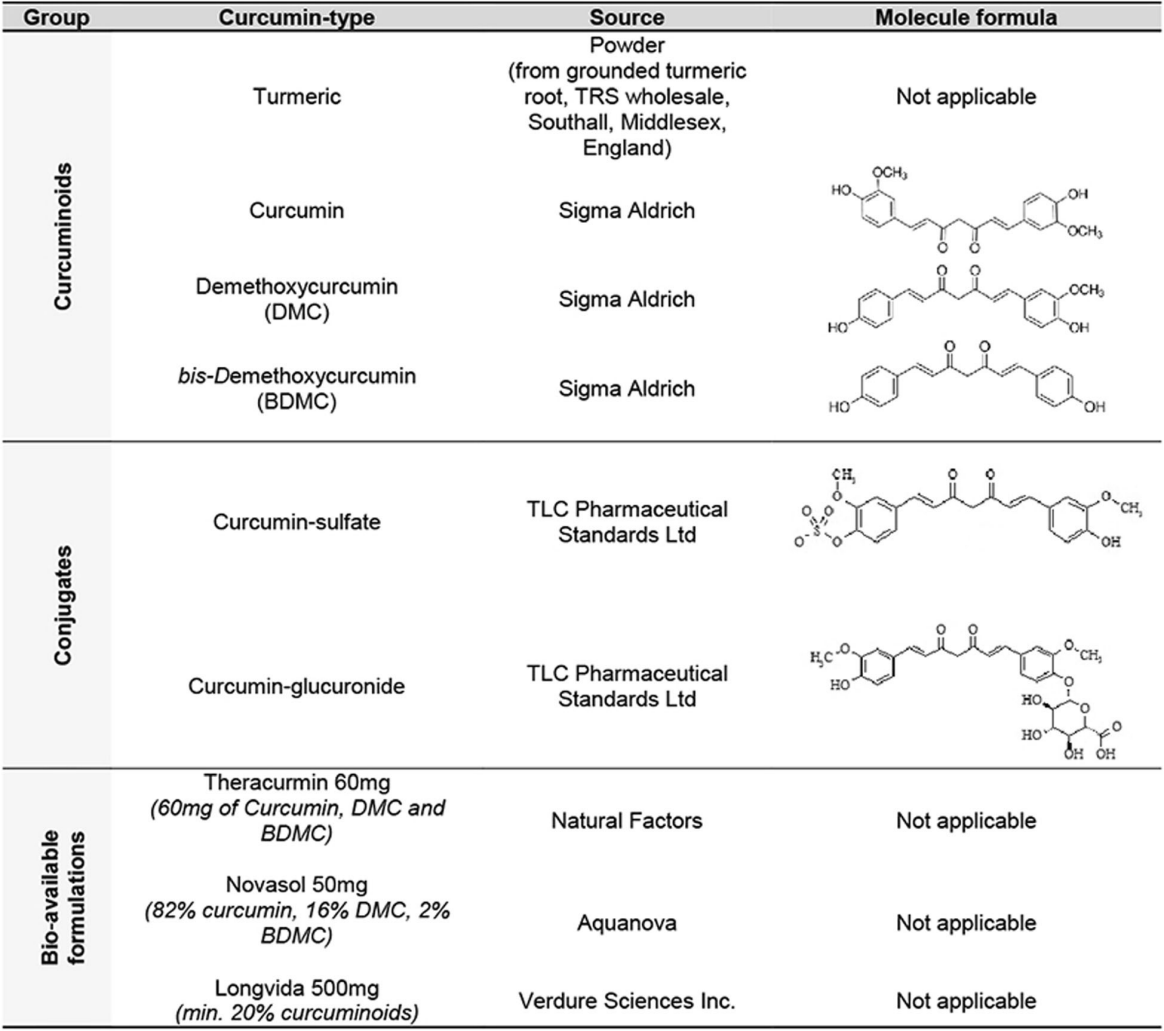
Curcuminoids, conjugates and bio-available forms of curcumin used for staining

### Combined curcumin staining and immunohistochemistry

Sections were deparaffinized and rehydrated, followed by blocking of endogenous peroxidase and antigen retrieval as described above. Subsequently, curcumin staining was performed, sections were washed in PBS, and immunohistochemistry was performed using anti-phosphorylated tau (anti-pTau, AT8,1:800) and anti-Aβ (IC-16, 1:400). Incubation was performed overnight, followed by washing in PBS. Secondary fluorescently labeled antibody (Goat Anti-mouse 594, Life Tech, 1:250) was incubated for 1.5 h, followed by washing in PBS. Sections were counterstained with 4′6-diamidino-2-phenylindole (DAPI) and covered with TBS/Glycerol.

### Imaging

IHC sections were imaged by an Olympus BX41 microscope using Leica Application Suite (LAS) AF lite software (Wetzlar, Germany). Fluorescently stained slides were imaged by a Leica DMi8 fluorescence microscope using LAS AF lite and ImageJ software (National Institue of Health, USA).

## Results

### Curcumin binding in control and AD brain tissue

**Fig. 1:**
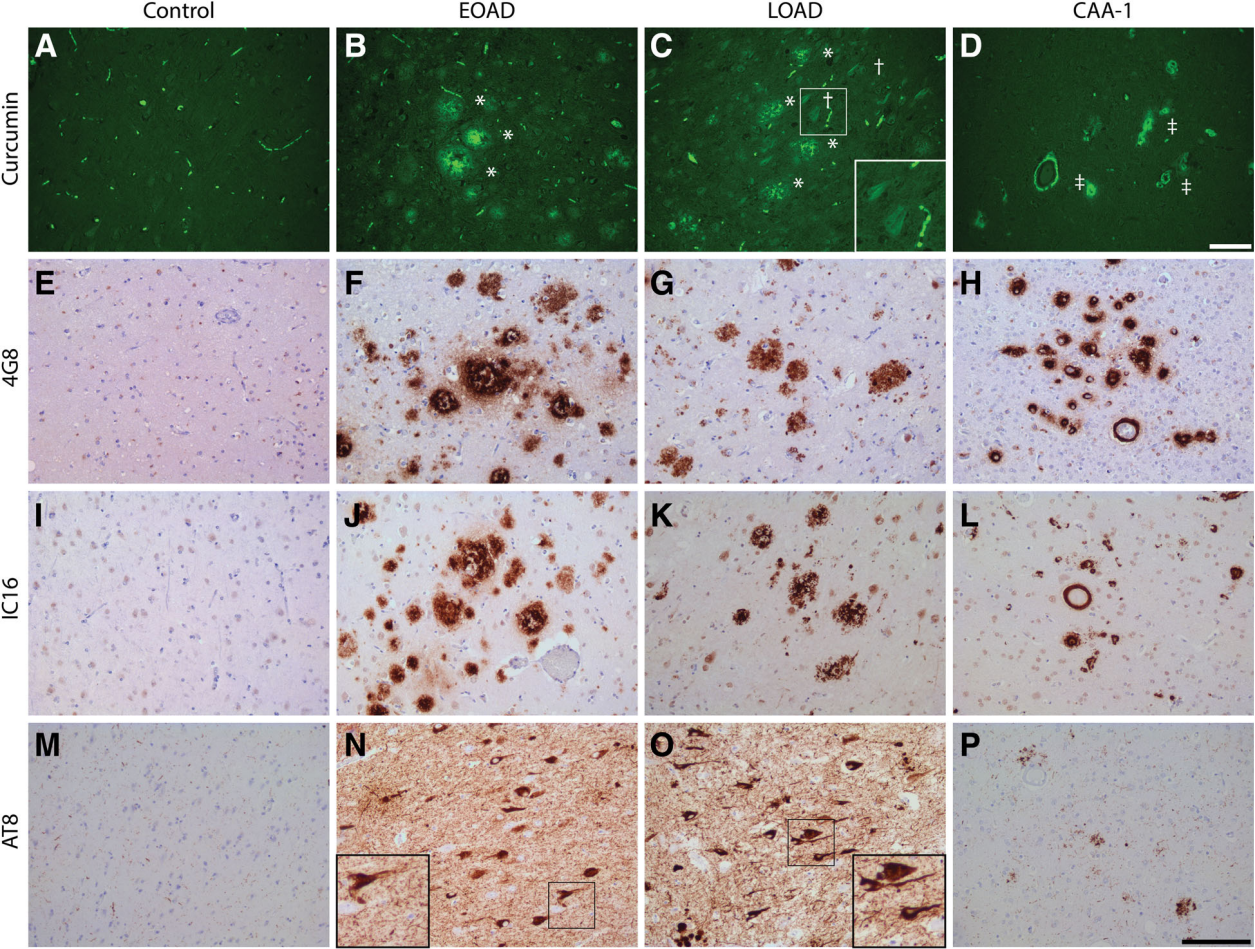
Detection of AD pathological hallmarks using curcumin. Curcumin staining of AD pathological hallmarks in early and late onset AD cases and AD cases with CAA type 1 (CAA-1) (**a**-**d**). Immunohistochemical DAB stainings for amyloid-beta (4G8 and IC-16) (**e**-**l**) and phosphorylated tau (AT8)(**m**-**p**) are shown for reference. Hippocampal blocks were used for AD-cases, while occipital blocks were used for CAA cases. IHC and fluorescent stainings were performed on the same brain region. Scale bars 100 μm. * = plaque, † = neurofibrillary tangle, ‡ = cerebral amyloid angiopathy. Abbreviations: EOAD = early onset Alzheimer’s disease, LOAD = late onset Alzheimer’s disease

In general, it was observed that curcumin binding on human brain tissue presented no background staining and auto-fluorescent signals in neurons. In brain tissue of EOAD, LOAD and AD cases with CAA (CAA type 1), curcumin strongly bound to amyloid plaques and CAA (Fig. 1). Curcumin staining revealed different types of plaques. We observed intense staining of primitive/compact plaques and moderate staining of the cores in cored plaques. In AD cases, neurofibrillary tangles (NFTs) showed a weak detection by curcumin. Adjacent sections were assessed by immunohistochemistry for the presence of Aβ and pTau in a corresponding area, which confirmed the presence of these pathological structures.

**Fig. 2:**
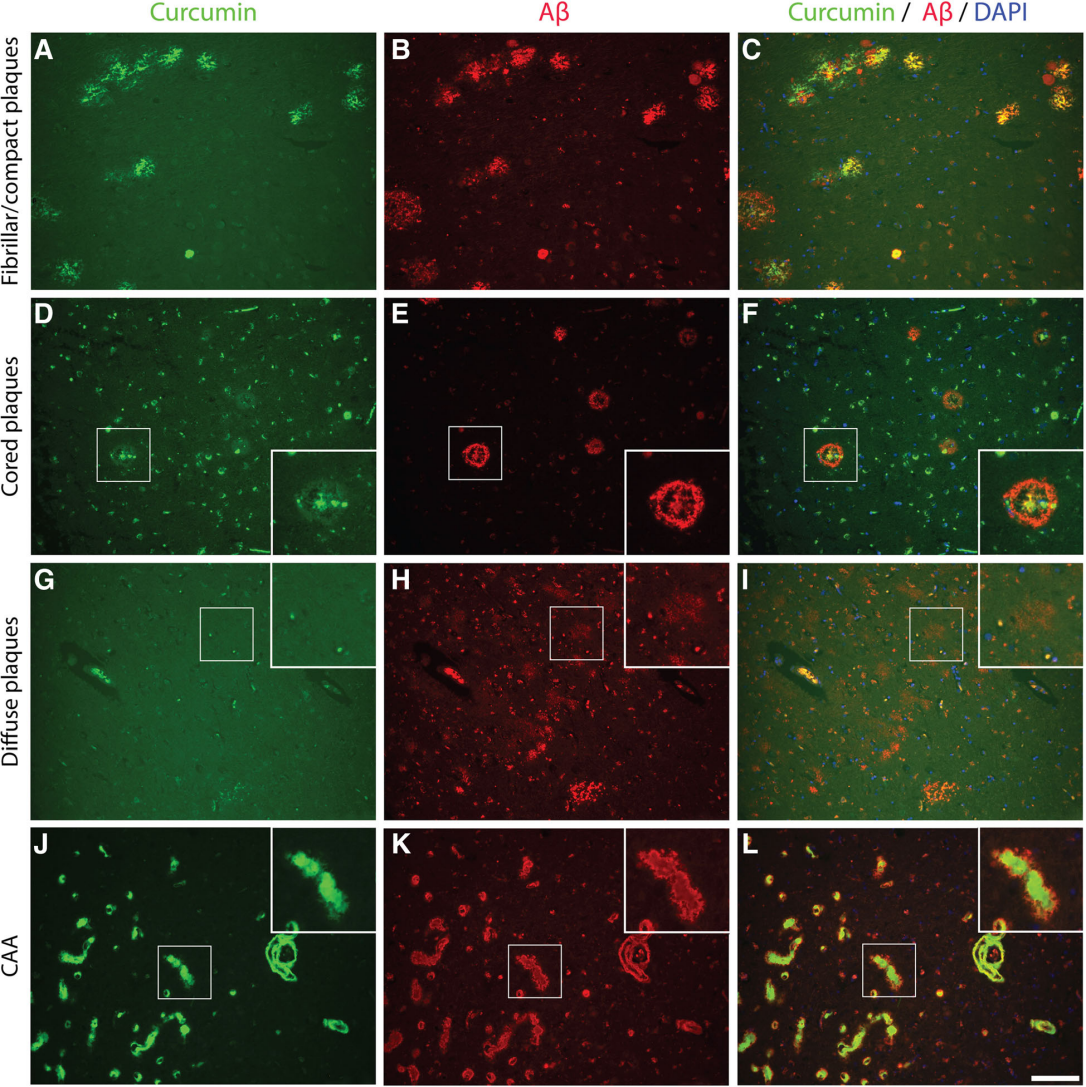
Co-labelling using curcumin and anti-amyloid beta (Aβ) in Alzheimer brains. Curcumin co-labelling with anti-amyloid-beta using IC-16 in Alzheimer brains (hippocampus (**a**-**c**), temporal cortex(**d**-**i**), occipital cortex(**j**-**l**)) to show overlap and differences. Scale bars 100 μm. Abbreviations: CAA = cerebral amyloid angiopathy, DAPI = 4′,6-diamidino-2-fenylindole

**Fig. 3:**
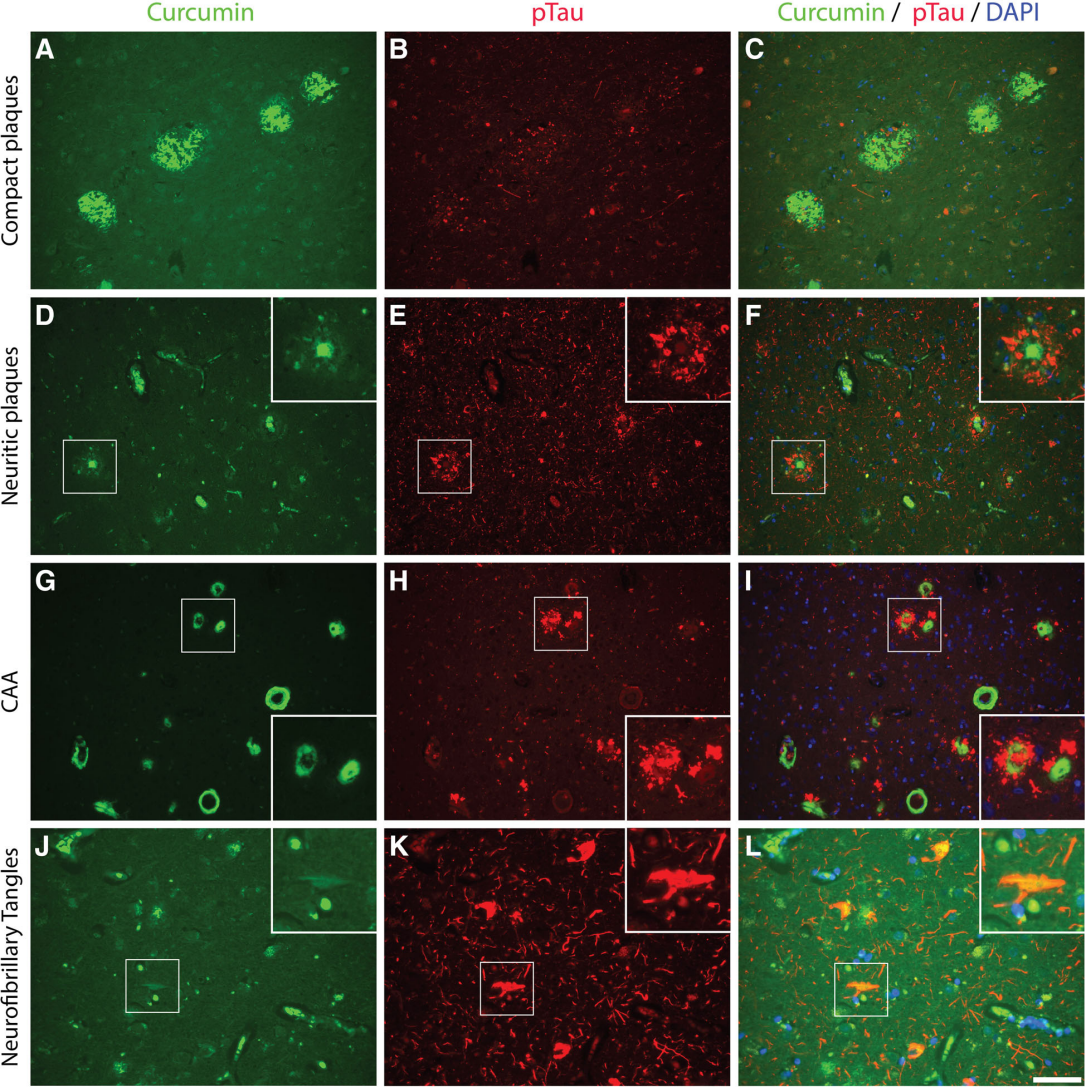
Co-labelling using curcumin and anti-phosphorylated Tau in Alzheimer brains. Curcumin co-labelling with anti-phosphorylated tau using AT8 in Alzheimer brains (hippocampus(**a**-**c**), temporal cortex(**d**-**f** and **j**-**l**), occipital cortex(**g**-**i**)) to show overlap and differences. Scale bars A-I 100 μm. Scale bars J-L 50 μm. Abbreviations: CAA = cerebral amyloid angiopathy, DAPI = 4′,6-diamidino-2-fenylindole

The binding of curcumin to these pathological structures was investigated by co-stainings for curcumin and Aβ (IC-16) or pTau (AT8). Primitive/compact plaques showed almost a complete overlap of curcumin signal and immunodetection by Aβ (Fig. 2). Classic cored plaques presented co-labeling of the core, while the corona of these plaques were immuno-labelled by anti-Aβ and not by curcumin. No binding of curcumin was observed in diffuse Aβ deposits. CAA affected blood vessels were stained most intensively by curcumin and showed a complete overlap with the detection of CAA by Aβ. While compact plaques showed moderate neuritic changes, as presented by anti-pTau immuno-labeling, no co-labelling of anti-pTau and curcumin was present in these plaques (Fig. 3). Neuritic cored plaques showed a strong immunodetection of pTau around the core. The core of neuritic plaques showed a strong binding of curcumin while co-labelling with anti-pTau was absent. A strong binding of curcumin was observed in CAA, however, no co-labelling with anti-pTau, observed around CAA affected capillaries, was detected. Co-labeling with anti-pTau confirmed that curcumin weakly detects NFTs.

### Curcumin does not bind to non-AD pathology

**Fig. 4:**
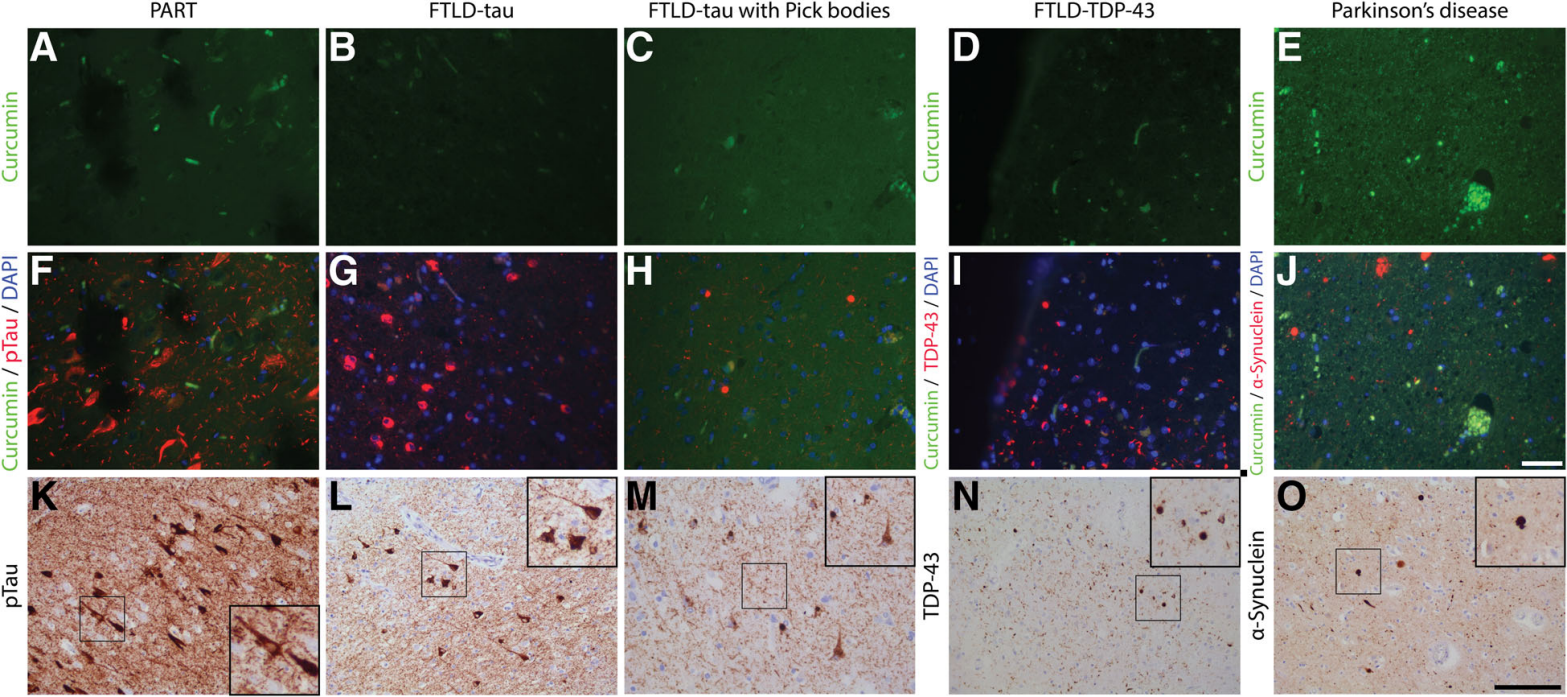
Curcumin binding in non-AD pathologies. Curcumin staining of PART, FTLD-tau, FTLD-TDP-43, and PD cases (**a**-**e**). Fluorescent co-stainings for comparison of curcumin and tau (FTLD-tau), TDP-43 (FTD-TDP-43), and α-synuclein (PD) (**f**-**j**). Immunohistochemical DAB stainings for tau (AT8), TDP-43 (pTDP-43) and α-synuclein (LB-509) are shown for reference (**k**-**o**). Scale bars **a**-**j** 50 μm. Scale bars **k**-**o** 100 μm. Abbreviations: DAPI = 4′,6-diamidino-2-fenylindole, FTLD-tau = frontotemporal lobe degeneration with tau pathology, FTLD-TDP-43 = frontotemporal lobe degeneration with TDP-43 pathology, PART = primary age related tauopathy

We investigated whether curcumin showed binding to protein inclusions or aggregates in non-AD pathologies. PART is characterized by the presence of NFTs and tau pathology in the absence of amyloid deposits [8, 19]. In cases with PART no specific binding of curcumin was observed. Co-labeling and single IHC with anti-pTau confirmed the presence of tau pathology and NFTs in the tissue section. In PART no binding of curcumin to NFTs was observed. Next, we assessed the binding of curcumin to tau inclusions in different FTLD-tau cases. Inclusions that were positive for pTau in cases with a MAPT P301L mutation, or in a case with sporadic Pick’s disease, did not show binding of curcumin. Also inclusions positive for TDP-43 in FTLD-TDP, and α-synuclein containing inclusions in PD and DLB were not detected by curcumin (Fig. 4).

### Staining of curcumin isoforms, conjugates and bio-available forms

**Fig. 5:**
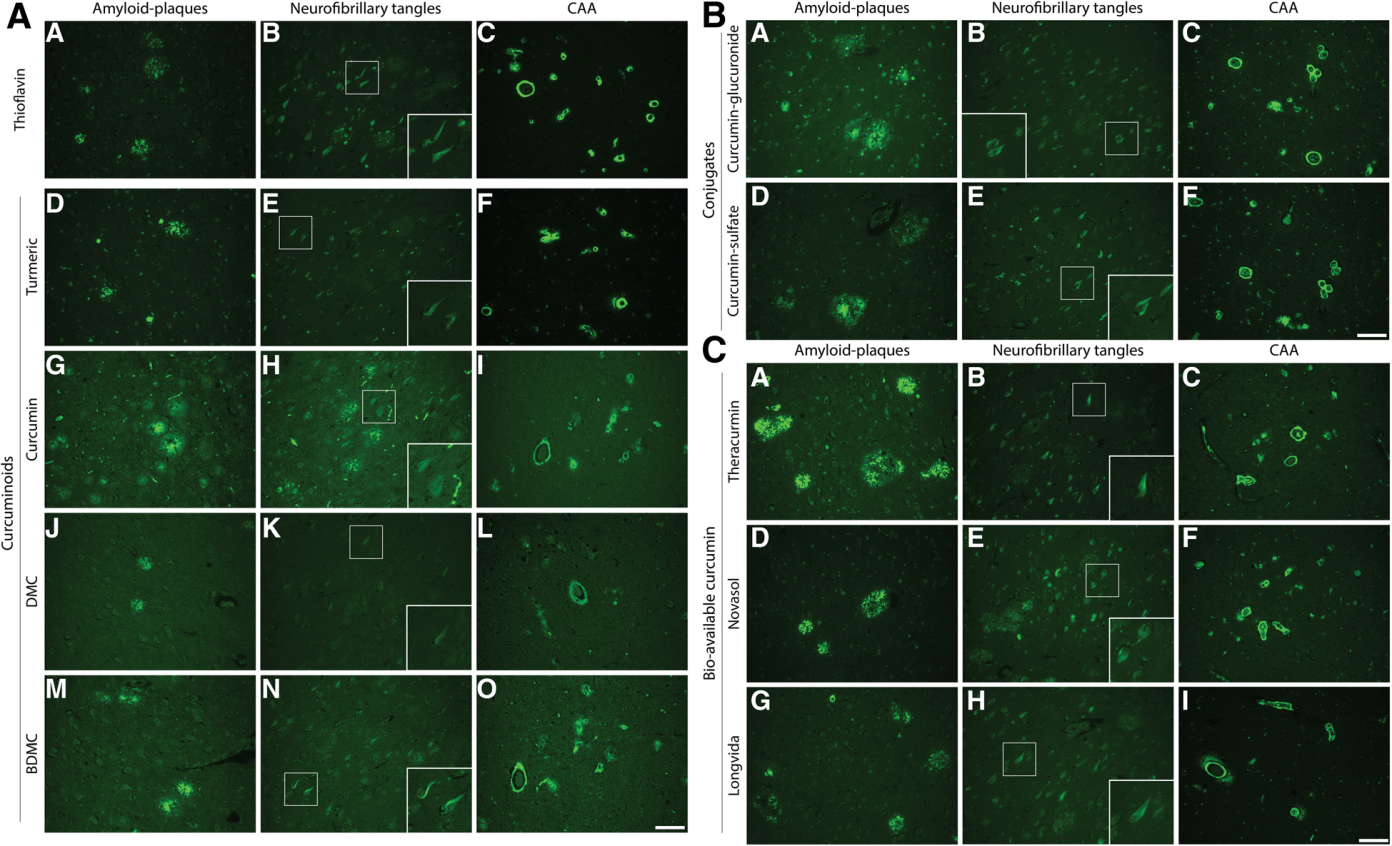
Staining of different curcumin forms to amyloid plaques, neurofibrillary tangles and **CAA.** Staining of amyloid-beta plaques, neurofibrillary tangles and CAA with Thioflavin-S and curcuminoids (**a**), conjugates (**b**) and bio-available forms (**c**). Scale bars 100 μm

In order to translate above findings to a possible in-vivo biomarker assay we assessed binding properties of different curcuminoids (DMC, BDMC), conjugates, and bio-available forms in AD cases. Given curcumin’s chemical similarities with Thioflavin, sharing an aromatic ring, we compared stainings to Thioflavin-S staining (Fig. 5). An overview of staining of different curcumin forms and Thioflavin-S to plaques, CAA and NFTs is given in Table 4.

**Table 4:**
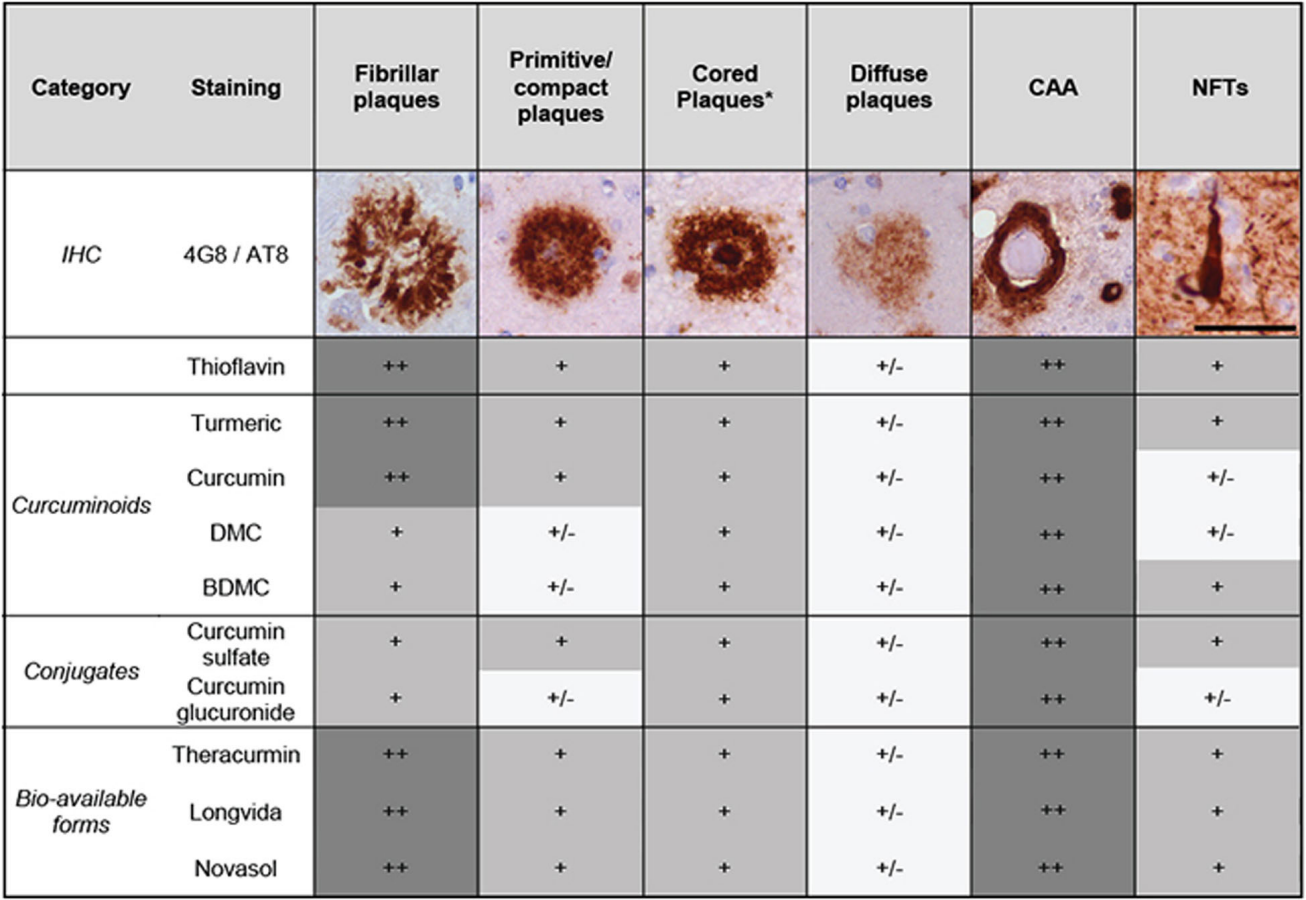
Overview of binding of different curcumin forms to different pathological deposits

Staining of different pathological deposits by different curcumin forms was qualitatively compared for different plaque types, cerebral amyloid angiopathy (CAA) and neurofibrillary tangles (NFTs). Immuno-histochemical (IHC) stainings for amyloid-beta (4G8, plaques and CAA) and phosphorylated tau (AT-8, NFT’s) are shown for reference. Scalebar 50 μm

++: clearly visible, +: visible, +/−: visible with appreciation of morphology. *The staining of the core of cored plaques rather than the corona was assessed here

Abbreviations: *BDMC* bis-demethoxycurcumin, *DMC* demethoxycurcumin

First, as turmeric also contains two isoforms: DMC (≈17%) and BDMC (≈3%), we assessed their binding. Similar to Thioflavin-S and curcumin, both isoforms bound amyloid plaques and CAA and showed weak staining of NFTs (Fig. 5a). Compared to Thioflavin-S, staining of fibrillar and primitive plaques by isoforms was slightly less apparent (Table 4).

Next, we assessed binding properties of curcumin conjugates (glucuronides, sulfates). As, curcumin is conjugated by the liver to make it water soluble, conjugates represent the majority of circulating curcuminoids. Curcumin-glucuronide and curcumin-sulfate bound to amyloid plaques and CAA (Fig. 5b). Curcumin glucuronide showed slightly less apparent staining of fibrillar, primitive/compact plaques and NFTs compared to Thioflavin-S (Table 4).

Finally, in order to translate above findings to clinically used oral bio-available formulations of curcumin we compared three bio-available formulations, namely Theracurmin, Novasol and Longvida. All three forms showed staining of primitive/compact plaques, the core of cored plaques, CAA and faintly stain NFTs, similar to Thioflavin-S (Table 4, Fig. 5c).

## Discussion

This study demonstrates that curcumin, curcuminoids, curcumin conjugates and bio-available formulations containing curcumin, bind to fibrillar amyloid in primitive/compact plaques, cored plaques and CAA in AD brain tissue. Curcumin isoforms and conjugates show few selective binding to NFTs in AD brain tissue and no binding to other neuropathological deposits, e.g. pTau and TDP-43 positive structures in FTLD and alpha-synuclein inclusions in PD and DLB. To the best of our knowledge we are the first to report that curcumin isoforms, conjugates and bio-available forms have similar staining properties as curcumin.

Comparing plaque types, binding of curcumin was more pronounced to amyloid deposits with a dense amyloid structure, as shown in Fig. 2 and Table 4. This is supported by co detection of curcumin with amyloid-beta in compact plaques while in contrast, diffuse plaques showed a lack of co-detection (Fig. 3). This indicates that curcumin binds preferably to dense amyloid structures, such as fibrillar plaques, rather than the amyloid beta peptide itself. The detection of amyloid by curcumin is comparable to the detection of amyloid by Thioflavin-S. This could be explained by the fact that curcumin and Thioflavin have chemical similarities, sharing a double aromatic ring [52].

Previous studies showed that curcumin binds Aβ-oligomers and fibrils in vitro [33, 44], Aβ-plaques in APPSWE/ PS1ΔE9, Tg2576 and 5xFAD transgenic mice [19, 24, 30, 45] and in post mortem human AD brain tissue [19, 31, 41, 45]. The latter human studies however assessed relatively small patient cohorts and did not assess other neurodegenerative diseases. Our findings in a well-characterized and large cohort of cases confirm earlier findings of specific binding of curcumin to fibrillar Aβ plaques, weak binding to NFT’s and rule out binding to pathological protein aggregates in other neurodegenerative diseases. Binding to fibrillary plaques might be of clinical relevance, as an increase of fibrillar plaques is associated with clinical progression to dementia [6]. In addition to curcumin binding to amyloid plaques, Mutsuga et al. described curcumin staining to CAA in larger vessels of an AD case as well as in various animals [39]. CAA can be subdivided in CAA type 1 (CAA-1) and CAA type 2(CAA-2) [47]. CAA-1 is characterized by Aβ presence in leptomeningeal and cortical arteries, arterioles, veins and venules as well as cortical capillaries, while CAA-2 on the other hand, affects leptomeningeal and cortical vessels with the exception of capillaries. In addition to earlier findings in CAA-2 by Mutsuga et al. we showed that curcumin also binds to CAA in capillaries in CAA-1 cases [39].

To the existing literature we added the comparison of the binding properties of different curcumin forms in human brain tissue, which is needed to translate findings for possible use in in-vivo diagnostic or therapeutic studies. The majority of curcumin and its isoforms are conjugated in-vivo and conjugated curcumin represents a large proportion of circulating curcumin [12]. To overcome poor bio-availability, delivery systems containing curcuminoids were developed. We showed that isoforms and conjugates and newly developed delivery systems of curcumin bind amyloid plaques and CAA and show comparable fluorescent properties as curcumin. For diagnostic studies this implies that all contents of newly developed delivery systems, including curcumin, DMC an BDMC and secondary formed curcumin conjugates harbor amyloid binding properties and could yield diagnostic purpose. Newly developed delivery systems, e.g. micelles, solid lipid nanoparticles and liposomes, did not show to affect amyloid binding properties. Similar binding properties could also indicate a therapeutic role of isoforms and conjugates. Previous studies showed that curcumin reverses existing amyloid pathology and associated neurotoxicity in a mouse model [9, 53]. If conjugates harbor the same functional effects on amyloid formation and related neurotoxicity is unknown. More research is therefore needed to assess possible functional effects of isoforms and conjugates on AD pathology.

Strengths of our study are the well-described patient cohort including various neurodegenerative features, thorough assessment of different curcumin forms and comparison with commonly used neuropathological IHC. Our patient cohort consists of a fair amount of well described AD-cases, cases with other neurodegenerative conditions as well as healthy controls, while previous studies assessed binding properties of curcumin mostly in animal models. Applying commonly used neuropathological IHC-staining we directly compared curcumin with established staining methods, which allows assessment of its specificity towards pathological structures. A limitation of our study is that the use of high concentrations curcumin in post mortem staining might not reflect concentrations that could be reached in-vivo. Although turmeric, with a mass percentage of only ≈3% curcuminoids, showed similar staining patterns and intensity as pure curcumin it should be noted that clinically reached concentrations might be unable to effectively bind pathology. Future studies might therefore test the minimal concentration needed to label pathology.

Secondly, we tested a wide variety of curcumin forms, however not synthesized forms of curcumin (e.g. CRANAD-28). CRANAD-28 might have amyloid plaques and CAA binding properties and beneficial blood brain barrier penetration, yet is not applicable for human use [54]. Lastly, as we focused on isoforms and conjugates representing the majority of circulating curcumin we did not test reduced forms of curcumin. Future studies might test binding properties of synthesized and reduced forms of curcumin.

The retina, as a protrusion of the central nervous system, might reflect neurodegenerative disease and is therefore of interest as a target for in-vivo fluorescent imaging both in ophthalmology and neurology. Previously, Cordeiro et al. visualized apoptotic cells in glaucoma patients with fluorescent imaging and showed a successful example of fluorescent molecular imaging in the retina [3]. Others claimed amyloid presence in both post mortem and in-vivo retinas of AD patients, visualized with curcumin [22–24]. This finding is controversial however, as other groups were unable to replicate post mortem detection of retinal amyloid [13, 46, 51]. Our results support the notion that, if fibrillar amyloid or amyloid angiopathy are present in the retina in AD, curcuminoids might be used as labeling fluorophore for non-invasive fluorescent retinal imaging.

## Conclusion

In conclusion, curcumin, its isoforms, conjugates and bio-available forms bind to fibrillar Aβ plaques and CAA, and faintly stain neurofibrillary tangles in post mortem AD brain tissue. They do not show binding in control brain or to specific structures observed in other neurodegenerative disease like FTLD and PD. As conjugates and bio-available curcumin forms show comparable binding properties, curcumin might be an interesting candidate for in-vivo diagnostics in AD like retinal fluorescent imaging.

## Abbreviations

AD: Alzheimer’s disease
Aβ: Amyloid-beta
BDMC: Bis-demethoxycurcumin
BMC: Demethoxycurcumin
CAA: Cerebral amyloid angiopathy
CAA-1: Cerebral amyloid angiopathy type 1
CAA-2: Cerebral amyloid angiopathy type 2
CSF: Cerebrospinal fluid
DAB: 3,3′ –diaminobenzidine
DAPI: 4′6-diamidino-2-phenylindole
DLB: Dementia with Lewy bodies
EOAD: Early onset Alzheimer’s disease
FTLD: Frontotemporal lobar degeneration
IHC: Immunohistochemistry
LOAD: Late onset Alzheimer’s disease
MRI: Magnetic resonance imaging
NBB: The Netherlands Brain Bank
NFTs: Neurofibrillary tangles
PART: Primary age-related tauopathy
PBS: Phosphate-buffered saline
PD: Parkinson’s disease
PET: Positron emission tomography
PiD: Pick’s disease
PM delay: Post mortem delay
pTau: Phosphorylated tau
TBS: Tris-buffered saline
TDP-43: TAR DNA-binding protein 43
